# Toughening of Epoxy Adhesives by Combined Interaction of Carbon Nanotubes and Silsesquioxanes

**DOI:** 10.3390/ma10101131

**Published:** 2017-09-25

**Authors:** Giuseppina Barra, Luigi Vertuccio, Umberto Vietri, Carlo Naddeo, Homayoun Hadavinia, Liberata Guadagno

**Affiliations:** 1 Department of Industrial Engineering, University of Salerno Via Giovanni Paolo II, 132-84084 Fisciano (SA), Italy; lvertuccio@unisa.it (L.V.); umbertovietri@gmail.com (U.V.); cnaddeo@unisa.it (C.N.); 2 School of Engineering, Kingston University, Roehampton Vale, Friars Avenue, London SW15 3DW, UK

**Keywords:** epoxy resin, adhesive, POSS, carbon nanotubes, single lap joints

## Abstract

The extensive use of adhesives in many structural applications in the transport industry and particularly in the aeronautic field is due to numerous advantages of bonded joints. However, still many researchers are working to enhance the mechanical properties and rheological performance of adhesives by using nanoadditives. In this study the effect of the addition of Multi-Wall Carbon Nanotubes (MWCNTs) with Polyhedral Oligomeric Silsesquioxane (POSS) compounds, either Glycidyl Oligomeric Silsesquioxanes (GPOSS) or DodecaPhenyl Oligomeric Silsesquioxanes (DPHPOSS) to Tetraglycidyl Methylene Dianiline (TGMDA) epoxy formulation, was investigated. The formulations contain neither a tougher matrix such as elastomers nor other additives typically used to provide a closer match in the coefficient of thermal expansion in order to discriminate only the effect of the addition of the above-mentioned components. Bonded aluminium single lap joints were made using both untreated and Chromic Acid Anodisation (CAA)-treated aluminium alloy T2024 adherends. The effects of the different chemical functionalities of POSS compounds, as well as the synergistic effect between the MWCNT and POSS combination on adhesion strength, were evaluated by viscosity measurement, tensile tests, Dynamic Mechanical Analysis (DMA), single lap joint shear strength tests, and morphological investigation. The best performance in the Lap Shear Strength (LSS) of the manufactured joints has been found for treated adherends bonded with epoxy adhesive containing MWCNTs and GPOSS. Carbon nanotubes have been found to play a very effective bridging function across the fracture surface of the bonded joints.

## 1. Introduction

Adhesively bonded joints have many structural applications in the transport industry and particularly in the aeronautic field. Compared to mechanical joints, they allow distributing the load over a much wider area, reducing fabrication costs, increasing fatigue resistance of the joints, and improving aerodynamics over bonded areas, which results in considerable weight saving. These advantages are applicable to both metallic and composite aircraft structures. In fact over 15% of the structural weight of aluminium airplanes can be saved by using bonding techniques as opposed to using rivets and bolts [1,2]. Also with bonded structures the maintenance cost is reduced due to the extension of the inspection interval, e.g., in Airbus A350, in comparison with the previous generation aircraft Airbus A330, direct maintenance costs are reduced by 15% [3]. Moreover, the upward trend in using hybrid systems featuring fiber reinforced polymer composites (FRPC) as structural materials in aeronautic and commercial transport vehicles requires the ability to join dissimilar materials. In this context, the use of polymeric adhesives as an alternative solution to using rivets/bolts (which create stress concentration and/or corrosion initiation), and welding is of potential industrial interest. During the welding, the heating of adherends can lead to significant thermal stresses due to the difference in the coefficient of thermal expansion (CTE) between dissimilar materials (for instance the base metal, the welding material, and the composite). These induced stresses can result in the loss of dimensional stability (shrinkage), or delamination in these parts [4,5]. However, the design of safe and cost-effective bonded joints is still a major challenge, and there is the need of a good understanding of the influence of material and geometric parameters on the joint’s strength [6].

The quality of the adhesive bonded joint depends on different factors such as the suitable surface pretreatment of the adherends, the choice of the adhesive, the joint design, and the service conditions.

In metal-metal bonding, adhesives are active only on the molecular surface layer that forms the joint interface and on any surfaces contained in the porosity of the metal itself; consequently, adhesives for bonding metals are studied to bond metal–oxide. Aluminium (Al) is an almost ideal substrate for adhesives. However, the corrosion resistance of Al, as well as the durability of the joints made with epoxy adhesives, is very dependent on the type of Al alloy used. Bonds made with relatively corrosion-resistant 6061-T6 Al alloy will last about four times longer than equivalent joints made with the 2024-T3 Al alloy when exposed to marine environments. Fortunately, with the proper combination of surface treatment and adhesive, the adverse effect on durability to aggressive environments can be minimized [7,8]. In addition, Al has a very reactive surface, and a cohesively strong oxide forms almost instantaneously when a freshly machined Al surface is exposed to the atmosphere. Fortunately, the oxide is extremely stable, and it adheres to the base metal with strength higher than that could be provided by most of available adhesives. The strength of the epoxy-bonded Al joint can be improved by cleaning the surface to remove contaminants or by converting the existing surface to a new surface that may be more consistent. In the case of the Al surface, chemical conversion can also protect the base metal from corrosion and enhance the durability of the bonded joint to various service environments. Between the different chemical surface treatments, chromate conversion coating on Al constitutes an effective way to enhance the surface bondability and also to improve the durability of the joint and the corrosion resistance of the interface.

Newly modified adhesives characterized by high adhesion strength, lower curing temperature, high fracture toughness, and other functionalities, such as electrical conductivity and heat/flame retardant resistance, are of practical interest [9].

In order to take the benefits that nanotechnologies offer in the field of structural composite [10], many researchers are working on the development of new multifunctional adhesives for structural applications. The strategy is based on appropriate epoxy resins nano-modified by carbon nano-forms, which enable it to increase the fracture toughness of the adhesive and hinder the insulator properties of the resin if employed beyond their Electrical Percolation Threshold (EPT) [11,12,13,14,15,16,17,18,19,20]. This strategy allows us to simultaneously optimize the efficiency of the joints and preserve the conductivity of the lightweight materials that could be also able to provide self-sensing capabilities, as well as good lightning strike protection in the joints [21,22]. Still, different types of hybrid nanofillers could be used in synergy with carbon nanostructured forms into the epoxy matrix, which not only improve the flame resistance and the thermal and photo-oxidative stability [23,24,25,26], but also enhance the adhesive strength and toughness [27,28]. Furthermore, the addition of hybrid nanofillers gives also a further benefit of balancing the increase in the viscosity of carbon nanofilled epoxy formulations [24]. For this purpose, the recent reinforcement of polymer systems with nano-sized inorganic clusters, Polyhedrical Oligomeric SilSesquioxane (POSS), has been given considerable attention. POSS reagents combine a hybrid inorganic-organic structure, having dimensions comparable to those of most polymeric segments or coil that can be bound to the polymer, leading to the reinforcement of the system on molecular level [24]. Recent studies have also shown that incorporated POSS in the polymer may have the potential to behave both like a filler particle and/or a plasticizing molecule, depending on the degree of dispersion in the polymer [29]. In addition, the incorporation of an appropriate amount of POSS in an adhesive can effectively enhance its mechanical performance. In the study shown in Ref. [30], the adhesive paste was applied to the aluminium bars adherends, which were preliminary degreased with trichloraethane and etched in Nochromix/sulfuric acid solution for 30 min, and then tested in single lap-bonded joint specimens. The authors observed that when using POSS with a concentration below 3 wt %, thermodynamically incompatible components form a loose transition region, which promotes a chain relaxation process and toughening of the adhesive. When POSS concentration was increased to 5 wt %, crystallization occurred over a large scale, leading to an increased glass transition temperature. This led to a decrease in adhesive bond line deformability and limited the further enhancement of adhesive performance. Also Jones et al. [31] studied thermal and mechanical properties of nanocomposites containing epoxy resin and POSS. The tensile test results indicated that 5 wt % loading of POSS in epoxy resin constituted the system with the highest improvement in tensile strength and elastic modulus.

A different approach was adopted by Dodiuk et al. [32,33] in studying the effect of the toughening of epoxy adhesives by POSS. They demonstrated that the design of new nanoscale-tailored adhesives with multifunctional properties required the appropriate selection and modification of the polymer matrix composition, the nanofiller type, and the control of the interactions at the interphase. In their studies, the shear and peel strengths of selected POSS epoxy formulations were characterized. In comparison with the neat epoxy formulation, an increase in shear strength of 20% was obtained for the selected POSS/epoxy formulations. Furthermore, a significant increase in peel strength was reported when the functionalities of the POSS were compatible with the epoxy system.

The aim of the current work is to study the effect of carbon nanotubes and POSS on the mechanical properties of the adhesive bonded aluminium joints, based on a TGMDA epoxy formulation containing neither toughening, such as elastomers, nor other additives typically used to provide a closer match in the coefficient of thermal expansion (CTE) between the adhesive and the adherends, such as aluminium or aluminium oxides powders. The reason why a blank formulation has been used was to discriminate only between the effects of the addition of the above-mentioned components. In addition, to establish the properties of nanofilled adhesives for aeronautic applications able to hinder both the insulating properties and poor flame resistance of epoxy resins, a different approach in preparing an adhesive was adopted: carbon nanotubes were dispersed above EPT [34,35,36], together with POSS compounds at the concentration of 5 wt %, into the epoxy resin. DodecaPhenyl Oligomeric Silsesquioxanes (DPHPOSS) and Glycidyl Oligomeric Silsesquioxanes (GPOSS) were used for this purpose. The choice of these two types of POSS is the result of previous research activities [37,38] where authors assessed the compatibility of these types of POSS molecules with a well-studied tetrafunctional epoxy resin, and also the effects of these nanofillers on the thermal stability and flame retardant properties of the resin [39]. In particular, the epoxy resin employed in developing the adhesive paste has proven to be very effective for improving nanofiller dispersion due to a decrease in the viscosity [40,41,42]. Bonded single lap joints (SLJs) were made using aluminium alloy T2024 adherends, whilst some of the adherends were treated with Chromic Acid Anodisation (Alodine 1200S). Then, the effect of different chemical functionalities of POSS and the synergetic effect between the MWCNT and POSS combination on adhesion strength were evaluated, taking also into account the benefits due to the surface treatment of the adherends. It has been found that treating the adherends with Alodine chromic anodization and modifying the epoxy adhesive with both GPOSS and MWCNTs enhance the adhesion shear strength substantially.

## 2. Materials and Methods

### 2.1. Materials for Adhesive Formulations

Epoxy resin- The epoxy matrix was prepared by mixing the epoxy precursor TGMDA (epoxy equivalent weight 117–133 g/eq), with an epoxy reactive monomer 1,4-butanedioldiglycidylether (BDE) that acts as reactive diluent. These resins, both containing epoxy functionality, were obtained from Sigma-Aldrich (Milan, Italy). The epoxy mixture was made by mixing TGMDA with BDE monomer at a concentration ratio of 75:25 wt % epoxide to flexibilizer. In particular, the use of 25 wt % loading of reactive diluent has been chosen to reduce the viscosity of epoxy resin and hence to improve the nanofiller dispersion.

**Figure 1:**
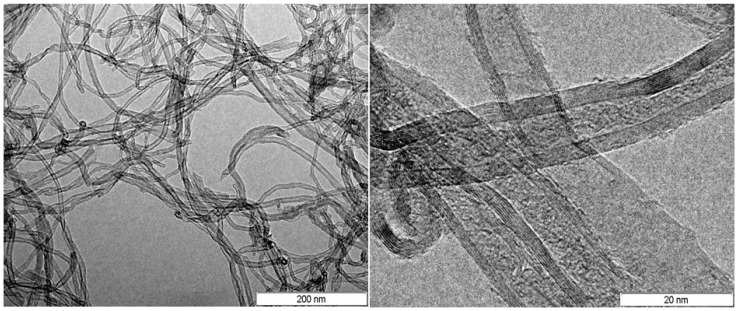
High Resolution Transmission Electron Microscope (HR-TEM) images of multi-wall carbon nanotubes (MWCNTs) at two different magnifications.

Carbon nanotubes- MWCNTs (3100 Grade) were obtained from Nanocyl S.A. and characterized via High Resolution Transmission Electron Microscope (HR-TEM) using a Jeol 2010 LaBa_6_ microscope operating at 200 kV (Jeol Ltd, Tokyo, Japan). MWCNTs were dispersed in ethanol by ultrasonic waves for 30 min. The resultant suspension was dropped on a copper grid (holey carbon). HR-TEM micrographs of MWCNTs at two different magnifications are shown in Figure 1.

The diameter of MWCNTs ranges from 9 nm to 30 nm, the distance between walls is approximately 0.35 nm, and the number of walls in nanotubes varies from 4 to 11. Nanotubes length is from hundreds of nm to few μm.

The specific surface area of the MWCNTs determined with the BET method is around 250–300 m^2^/g; the carbon purity is >95% with a metal oxide impurity <5%, as they were obtained from thermo-gravimetric analysis. An amount of 0.5 wt % of MWCNTs was used in nanocomposites.

POSS compounds- Two different POSS compounds were dispersed in the epoxy matrix: GPOSS, functionalized with eight oxirane groups for each cage, and DPHPOSS functionalized with phenyl groups. The structures of the used organic substituted POSS compounds are shown in Table 1.

Curing agent- The curing agent used was 4,4′-diaminodiphenyl sulfone (DDS). This hardener agent was added at a stoichiometric concentration with respect to all the epoxy rings (TGMDA, BDE, and GPOSS).

### 2.2. Adhesive Formulation Preparation

Epoxy blend (TGMDA and BDE) and DDS were mixed at 120 °C until complete hardener solubilization and then the mixture was cooled to 90 °C. Carbon nanotubes and POSS compounds were added simultaneously and incorporated into the matrix at 90 °C by using an ultra-sonication for 20 min. An ultrasonic device, Hielscher model UP200S (200 W, 24 kHz) (Hielscher Ultrasonics, Teltow, Germany), was used. All the tested adhesive compositions are shown in Table 2.

### 2.3. Adherends

**Figure 2:**
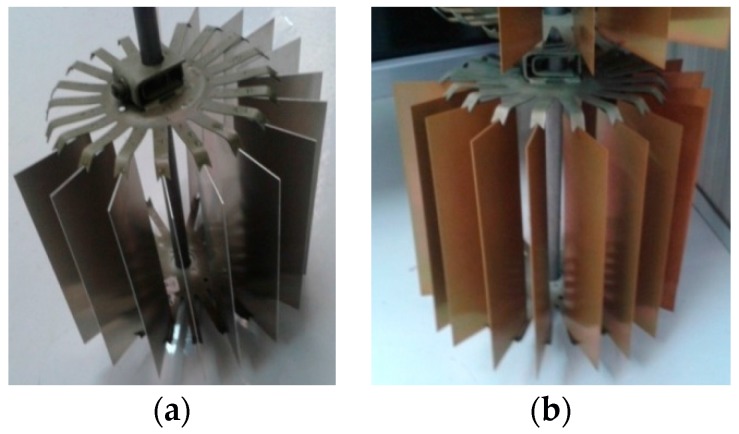
Aluminium plates: (**a**) before and (**b**) after chromic acid anodization with Alodine 1200S.

Metallic adherends from aluminium alloy 2024-T3 were supplied by TESI S.a.s. “Tecnologie E Servizi Innovativi” Cicerale (SA) (Italy). Five pairs of untreated aluminium adherends, for each adhesive formulation, having dimensions 101.6 mm × 25.4 mm × 1.26 mm, as indicated in the ASTM D1002-10 [43], were prepared. The other 25 pairs of metallic adherends with the same dimension have been exposed to a surface treatment usually used in European aeronautic industry, which is the chromic acid anodization (CAA) with Alodine 1200S. The surface treatment consisted of a degreasing step in a negligible abrasive weakly inorganic alkaline solution (pH ~ 9) followed by a strongly alkaline etching bath which removes the firmly attached oxide coatings and scrap marks. Afterward, a pickling process with oxidizing acids has been performed to remove another few μm of material to obtain a fresh surface. Finally, the aluminium slabs were subject to chromate conversion coating to increase the corrosion resistance. This last step results in formation of an amorphous hydrate oxide protective film which contains mainly complex chromium compounds and also little amounts of aluminium and copper compounds (see Figure 2).

### 2.4. Preparation of the Adhesively Bonded Single Lap Joints

**Figure 3:**
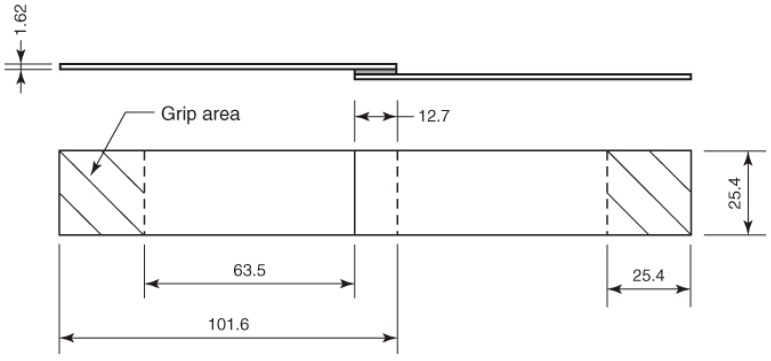
Scheme of test specimen for single lap joints test according to ASTM D 1002-10 standard. All dimensions in mm.

Both treated and untreated aluminium adherends were adhesively bonded to prepare joints according to the test specimen’s specification of ASTM D 1002-10, as shown in Figure 3. The overlap length was 12.7 mm; afterward, the joints were placed in a Carver press at 400 kPa and subjected to a two-step curing process: an initial step at moderate temperature (125 °C for 1 h) and the second one at higher temperature (180 °C for 3 h).

Table 3 summarizes the prepared samples for the single lap joint test. The thickness of the adhesive layer was controlled by 400 kPa pressure in the Carver press and measured by a digital Micrometer Screw Gauge (accuracy 0.01 mm). For each type of joint, five different samples were tested, and the adhesive thickness is indicated as the average ± standard deviation.

### 2.5. Experimental Studies

#### 2.5.1. Characterization of the Uncured Adhesives

Rheological measurements were performed using an AR 2000 TA Rheometer Instrument (TA Instruments Ltd, New Castle, USA). Parallel plates ϕ = 40 mm was selected as appropriate geometry and the gap was set at a value of 300 μm. Temperature sweeps from 70 °C to 140 °C were carried out at constant frequency of 1 Hz at strain % set at 5% within the linear viscoelastic region (determined from strain sweep test).

#### 2.5.2. Characterization of Cured Adhesives

Adhesive formulation described in Section 2.2 was poured into molds to prepare sample of appropriate dimensions for tensile and DMA tests. A two steps curing process has been performed: an initial step at moderate temperature (125 °C for 1 h) and the second one at higher temperature (180 °C for 3 h).

##### Tensile Test of Cured Adhesives

**Figure 4:**
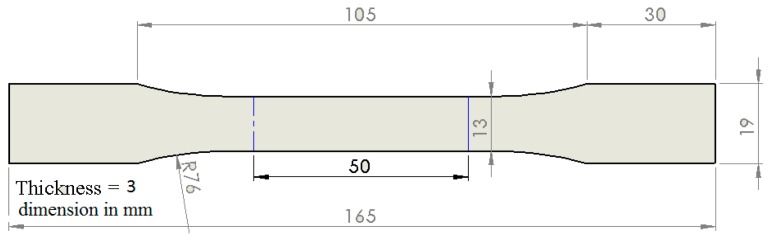
Specification of tensile test specimen according to ASTM D638-14. All dimensions in mm.

The elastic modulus of the adhesive paste was determined by the tensile test on bulk specimens according to ASTM D638-14 standard [44] (see Figure 4). Five dogbone samples for any formulation were prepared by casting the epoxy uncured formulation in dogbone shape molds. The five samples were tested for each compound in the tensile axial loading at a rate of 1 mm/min. For each case, Young’s modulus was obtained from measuring the slope of the stress–strain curve in the linear region [45].

##### Dynamic Mechanical Tests

Dynamic mechanical properties of the samples were obtained with a dynamic mechanical thermo-analyzer (Tritec 2000 DMA-Triton Technology, Grantham, UK). Solid samples with dimensions 2 × 10 × 35 mm^3^ were tested by applying a variable flexural deformation in three points bending mode. The displacement amplitude was set to 0.03 mm, whereas the measurements were performed at the frequency of 1 Hz. The range of temperature was from −90 °C to 315 °C at the scanning rate of 3 °C/min.

#### 2.5.3. Characterization of the Joints

##### Single Lap Joint Shear Strength Tests

Adhesively bonded single lap joints have been tested using an electro-hydraulic servo-controlled testing machine (Instron model 4301, Instron Co., Norwood, MA, USA) with 10 kN load cell. The specimens were placed in the testing machine and the load has been applied by setting a crosshead speed of 1.27 mm/min (0.05 inch/min), as indicated in the ASTM D1002-10. The self-aligning grips used were capable of securely grasping the specimen throughout the tests duration without allowing the specimens to slip. Furthermore, before performing experiments, the adherends in the proximity of the grips were marked out to allow the monitoring of possible slippage occurrences during the test. The apparent average shear strength of bonded lap shear joint was measured by dividing the maximum tensile load measured during the tests to the bonded area.

## 3. Results and Discussion

### 3.1. Viscosity Measurements

Viscosities of the uncured samples have been evaluated at temperatures close to the curing process in the range between 80 °C and 120 °C. In this range of temperatures, the measured viscosities for the DPHP-CNT/EP formulation containing both DPHPOSS and MWCNT are very similar to those obtained for formulation containing only carbon nanotubes (CNT/EP).

Table 4 summarizes the values of the viscosity at the specified temperatures within the curing process temperature range for the pristine epoxy, the epoxy formulation with 0.5% MWCNT, and the epoxy formulation with GP-CNT containing both GPOSS and MWCNT. Contrary to DPHPOSS, the GPOSS incorporation in epoxy strongly affects the viscosities. In fact, the formulation GP-CNT/EP containing both GPOSS and MWCNT has viscosities much lower than the CNT/EP formulation but higher than the viscosities of the unfilled epoxy formulation. This beneficial effect of the addition of GPOSS in lowering the viscosity of system is most probably due to the higher ability of GPOSS compared to DPHPOSS to react and to solubilize in the epoxy resin to produce a homogeneous continuous epoxy matrix, as shown by Raimondo et al. in previous published works [37].

### 3.2. Tensile Tests

**Figure 5:**
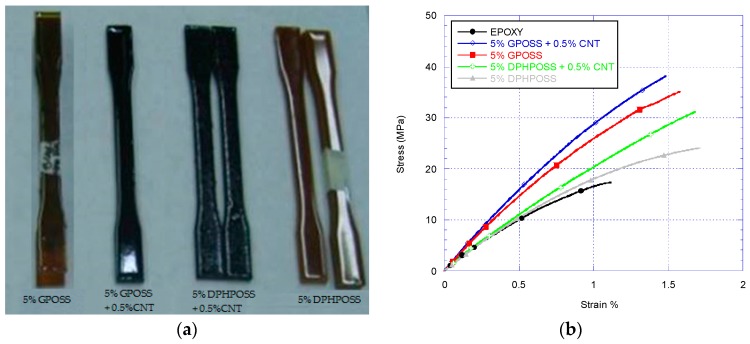
(**a**) Casted tensile specimens; (**b**) stress-strain curves of various epoxy nanocomposites.

**Figure 6:**
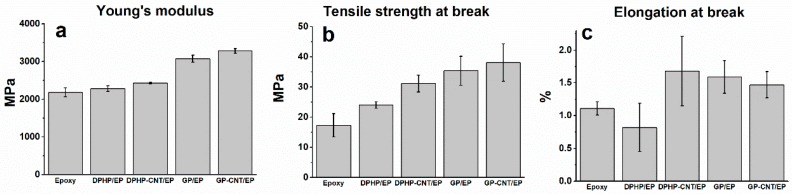
(**a**) Young’s modulus; (**b**) tensile strength and (**c**) elongation at break of the Epoxy, DPHP/EP, DPHP-CNT/EP, GP/EP, and GP-CNT/EP nanocomposites.

Figure 5a shows dogbone specimens casted from different nanocomposites and Figure 5b shows the stress-strain curves of various nanocomposites obtained from tensile tests. From the stress-strain diagrams, Young’s modulus, tensile strength, and elongation at break are extracted and the results are summarised in Figure 6. The epoxy with GP+CNT additives has the highest stiffness and strength in comparison with the other nanocomposites. The introduction of two different POSS leads to two different behaviours according to the physical properties and the structure of the incorporated POSS. From a mechanical point of view, specimen DPHP is very similar to the neat EPOXY in both the values of the Young’s modulus and the tensile strength at break. For this sample, the elongation at break instead increases to 1.71%. The poor solubility of DPHPOSS in epoxy resin causes the formation of non-continuity regions inside the matrix, which negatively affect the mechanical properties, especially the tensile strength. The increase of the elongation at break could be due to very weak reversible interactions between the twelve phenyl groups around the DPHPOSS cage, and the phenyl groups of the epoxy network. On the contrary, the introduction of GPOSS increases all the mechanical characteristics of the resin. DPHP, containing phenyl groups, is a low soluble powder in epoxy resin and gives rise to re-aggregation phenomena; as said before, GPOSS is instead a viscous liquid completely soluble in the epoxy resin used in this work [37]. Moreover, GPOSS has epoxy functionalities, which contribute to the matrix cross-linking reaction, allowing the formation of a continuous matrix system. Such a system, most likely, enables a load transfer distributed on the POSS units inside the epoxy matrix, which causes an increase in the Young’s modulus. The increase of the values at break point could be ascribed to an increase in the flexible chains in the matrix tracts due to the presence of linear side groups in the GPOSS cage. Because the epoxide network is brittle, the presence of soft dispersed-phase improves the strength at break. The introduction of MWCNTs in the formulation containing POSS has small effect on the mechanical parameters; the Young’s modulus and the tensile strength slightly increase but a slight decrease in elongation at break occurs. The low amount of added MWCNTs and the high mechanical performance of the pristine epoxy resin used, with a very high glass transition temperature, may be a justification of this behaviour. In contrast, the addition of MWCNTs to the formulation containing DPHPOSS leads to a significant increase in the tensile strength. As said before, DPHPOSS has twelve phenyl groups around the POSS cage, although it is not soluble and it does not react with epoxy, it may activate π–π stacking interactions with MWCNTs dispersed in the epoxy matrix. These interactions hinder and slow down the crack propagation with a consequent significant increase in the tensile strength at break.

### 3.3. Dynamic Mechanical Tests

**Figure 7:**
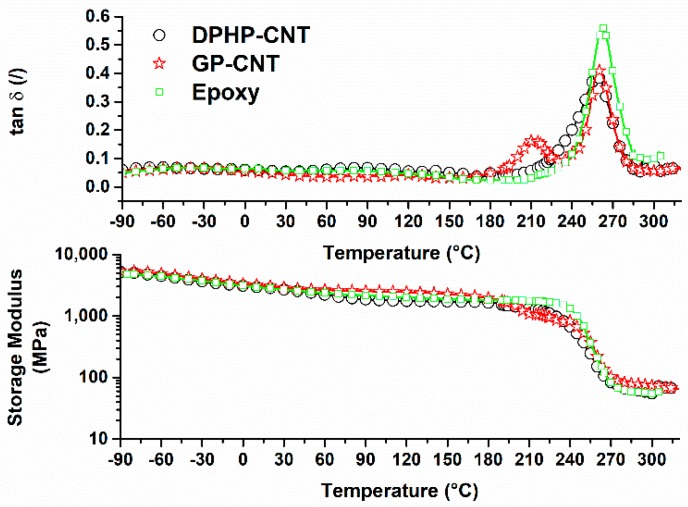
Results of dynamic mechanical analysis performed on cured Epoxy, DPHP-CNT/EP, and GP-CNT/EP samples.

DMA was performed on the cured samples. The storage modulus, *E*’ (MPa), and the loss factor (tan δ) of cured DPHP-CNT/EP and GP-CNT/EP, together with control sample Epoxy, are shown in Figure 7. Both the modified samples show storage modulus similar to the epoxy in the usual operational temperature range of structural materials up to 180 °C. The storage modulus slowly and progressively decreases until around 180 °C is reached. After this temperature, a pronounced decrease of the storage modulus in modified DPHP-CNT/EP and GP-CNT/EP nanocomposites indicating phenomena related to the glass transition is occurring. In the range of temperature between 180 °C and 300 °C, the tan δ spectrum of the sample DPHP-CNT/EP shows a wide peak centred at the value of 260 °C, while sample GP-CNT/EP presents two peaks: the first centred at 210 °C and the second centred at 260 °C. The peak of tan δ for pure epoxy is at 262.5 °C. A previous work made on GP-CNT/EP system [38] highlighted that the presence of MWCNT is responsible for a greater mobility of chains belonging to domains finely interpenetrated in the matrix, where the reversible hydrogen bonds determined by interaction between epoxy resin and nanocages of POSS compounds can provide unexpected cohesive forces.

Sample DPHP-CNT/EP, instead, behaves very similar to what has already been found in literature for pure epoxy resin; in this case, the inhomogeneity of the epoxy resin containing both DPHPOSS and MWCNT hinders the segmental motion and hampers the formation of a phase with higher mobility.

### 3.4. Single Lap Joint Shear Strength Tests

**Figure 8:**
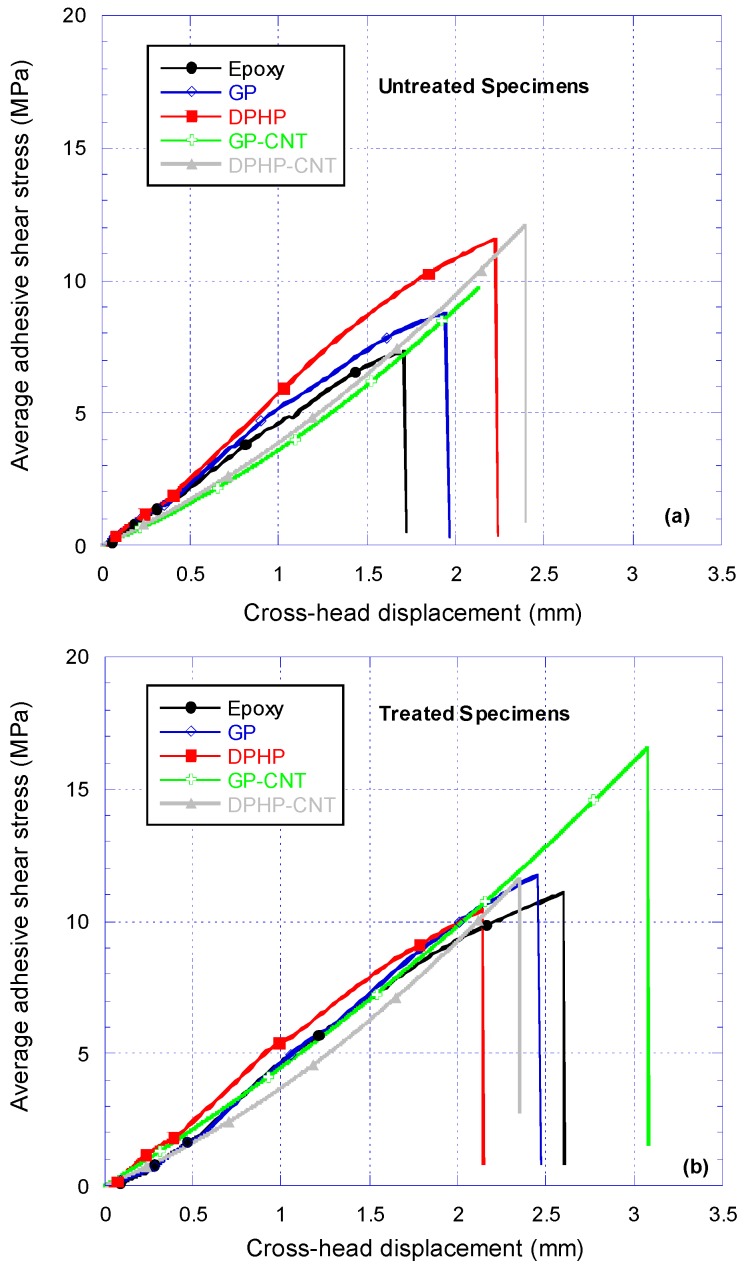
Single lap joint shear tests on joints made with (**a**) untreated and (**b**) treated adherends for various nanocomposite systems.

**Figure 9:**
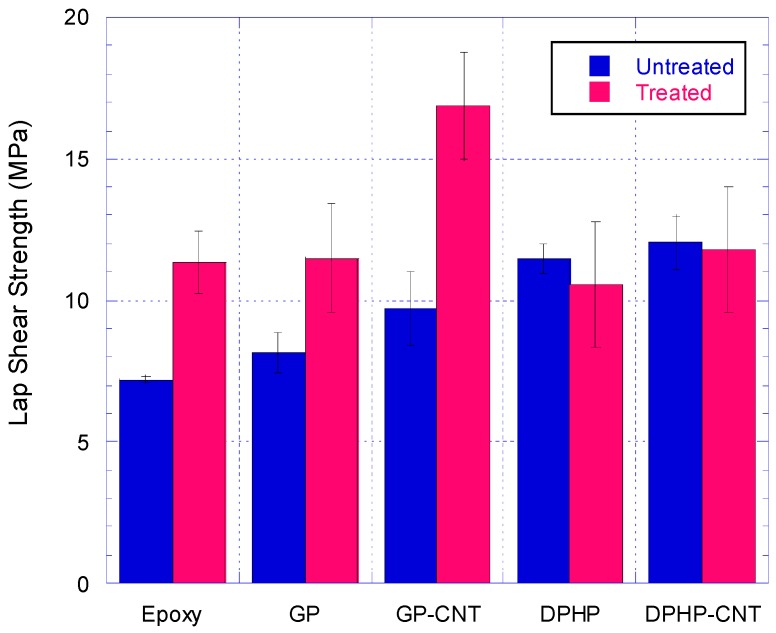
The results of single lap joint shear strength (LSS) tests on untreated and Chromic Acid Anodisation (CAA)-treated substrates bonded with epoxy, DPHP, DPHP-CNT, GP, and GP-CNT epoxy nanocomposites.

Lap shear strengths (LSS) were obtained by testing single lap joint specimens made according to the joints’ specification described in Table 3. The tests’ results are shown in Figure 8 and Figure 9.

#### 3.4.1. Discussion on Untreated Adherends SLJ Test Results

For untreated adherends, the introduction of POSS in the epoxy matrix led to an enhancement of the lap shear strength (LSS) of the joints. For these systems a further introduction of MWCNTs led to further improvement in the LSS values. For this series of coupons, the best results were obtained for DPHP systems. An analysis of the failure mechanism of the joints is surely important to elucidate the effective contribution of the introduction of components on the mechanical performances of the joints. In fact, for all the joints made with untreated aluminium adherends, the adhesive remained only on one of the substrates, indicating that these joints failed adhesively along the interface. The reinforcing effect obtained through the introduction of DPHP epoxy system, most likely, is due to the formation of a stronger adhesive–substrate interface. DPHP acts as a coupling agent, improving the interfacial wettability and chemical compatibility between the adhesive and the metallic substrate (aluminium oxide). Dodiuk et al. [32,46], studied the effect of the functional groups on the POSS cage on the shear strength of epoxy adhesive in aluminium joints. They observed that addition of small amount of functionalized POSS to neat epoxy adhesives, based on DGEBA, could even double the shear strength. In both the Dodiuk’s papers, three types of functionalization were investigated: Epoxycyclohexyl, Aminoethyl, and Octaphenyl. The best result in toughening of epoxy adhesives was obtained with the Octaphenyl functionalization. Octaphenyl and DodecaPhenyl functionalized POSS have the same functional group R (Phenyl) and only differ in the number of Si vertices. Both of them tend to crystallize, are poorly soluble in epoxy resin, and provide the best results in toughening the epoxy adhesives. POSS, which does not distribute homogeneously, tends to agglomerate in composites. Phase separation frequently occurs between POSS and monomer(s) before and during polymerization, depending on the chemical nature and functionality of the organic ligands in POSS cages. It is observed that the POSS-based composite has ultra-low friction and gradient distribution of POSS, which tends to enrich on the surface rather than inside [47,48].

Aluminium alloy T2024-T3 contains about 4% of Cu, but also 1.2–1.8% of Mg. The processing of this alloy to obtain aluminium sheets causes an increase of the magnesium concentration at the surfaces. Magnesium, in contact to the environmental conditions oxides to MgO and to Mg(OH)_2_, generates free metallic ions Mg^2+^. The surface of the alloy sheet will have, hence, a very complex composition with aluminium oxide and hydroxide; magnesium oxide and hydroxide; and carbonate and copper oxide. All the metals, and in particular the magnesium, are electron acceptors and may interact with the more electronegative oxygen at the bridge between two Si in the POSS cage or with the phenyl groups of the DPHPOSS, improving the wettability and the adhesion between the DPHPOSS-containing epoxy adhesive and the aluminium alloy. For untreated adherends, the addition of GPOSS in epoxy matrix does not lead to any significant enhancement of the adhesion. GPOSS, which does not have the phenyl electron donor functionality, and being soluble in the epoxy matrix and contributing via its epoxy functionalities to the matrix cross-linking, is frozen inside the epoxy network structure and is not free to interact with the electron acceptor metallic ions. For this reason, the adhesion properties of sample containing GPOSS are comparable to those of unfilled epoxy adhesives. The presence of CNTs causes a slight improvement, but not very significant in the limit of the experimental error. It can be seen from Figure 8a that addition of CNTs softens the nanocomposite and the stiffness of untreated SLJs is dropped below SLJs with nanocomposite without CNTs. However, for CAA treated specimen, as shown in Figure 8b, the stiffness of specimens with GP-CNT nanocomposite is as good as unmodified epoxy and GP specimens.

#### 3.4.2. Discussion on Treated Adherends SLJ Test Results

Chromic acid anodization (CAA) causes an increase of about 50% in LSS for the joints made with epoxy and GPOSS adhesive formulations. In contrast, for sample containing DPHP in this treatment does not lead to any significant enhancement.

The strong enhancement in the values of LSS of the joints composed of treated adherends and Epoxy, GP/EP, and GP-CNT/EP adhesive formulations with respect to the same joints with untreated adherends is undoubtedly due to a strong increase in adhesion forces at the interface between adhesive and the surface of the treated adherend. In the case of the joints with DPHP/EP and DPHP-CNT/EP, the adhesion forces at the interface are similar or even weaker with respect to those active between the adhesive and the untreated adherend. The reason for this different behavior can be understood considering the chemical nature of the surface of the treated adherend, as well as its morphological feature together with interactions at the interface.

The protection provided by CAA creates a thicker and denser aluminium oxide layer by anodizing, and leads to a porous structure where the bottom of the pores is blocked by the dense barrier layer. In a typical aerospace structure, pores are about 30 nm wide and the oxide is about 1 μm thick [8,49,50]. This treatment provides an extreme stable porous surface to be adhesively bonded with consequently improved adhesion properties, as it has been possible to observe for the joints bonded with epoxy and GP formulations. Recent studies made by Abrahami et al. [51] have demonstrated that pore size plays a role in determining the amount of resin penetration and also that the maximum peel strength is strictly dependent on pore size in the range of between 15 nm and 25 nm, and is independent of the oxide layer thickness. In our CAA treated SLJs, adhesives can penetrate in the CAA-generated oxide layer to create a well-wetted interface with the formation of a “micro-composite” interphase. This will reduce the local stress concentrations as reported in ref [52], because this region will possess an intermediate modulus between that of the relatively low-modulus polymeric adhesive and high-modulus aluminium alloy substrate.

Furthermore, the presence of GPOSS strongly contributes to creating cumulative effects in the attractive interactions between GPOSS nanocages and oxides on the surface.

Regarding the samples DPHP/EP and DPHP-CNT/EP bonded with the formulations containing DPHPOSS/EP, it should be considered that [32,46] the CAA treatment involves an intermediate deoxidizer step that, combined with the other acid treatment, allows for the complete removal of the magnesium from the surface. For DPHP/EP and DPHP-CNT/EP samples, the improvement on adhesion properties caused by the formation of the extremely stable surface is negatively counterbalanced by the depletion of the magnesium ions interacting with the more electronegative units of the POSS structure. For this reason, apparently the results do not show any improvement of the toughening of the epoxy adhesive, as can be seen from Figure 9.

Finally, the Alodine chromic anodization of the adherends of sample GP-CNT/EP, with adhesive composition containing both GPOSS and MWCNTs, causes a distinct improvement of the adhesion properties resulting in cohesive failure mode. The LSS almost doubles its value compared with the value obtained for the untreated sample. This important improvement is due to the combined effects of at least three different phenomena: (i) the development of a stable surface of the adherends; (ii) the better dispersion of CNTs in the matrix and filling of the adherends’ surface pores as a result of a decrease in viscosity of the epoxy adhesive; and (iii) the formation of a phase with higher mobility governed by hydrogen bonds. Regarding the second point, the lowering of the viscosity and the easier flow of the resin to fill the pores play a dominant role in building a stronger interphase leading to cohesive failure with the micro bridging of fracture surface by CNTs; concerning the third point, as previously observed by DMA of the analyzed adhesive formulations, the inclusion of MWCNTs in the formulation containing GPOSS determines a phase with a lower transition in the mechanical spectrum (centred at about 210 °C), indicating the presence of a phase with greater mobility of chain segments. MWCNT are responsible for forming in the resin a second phase characterized by different crosslinking density. GP-CNT/EP formulation will be hence characterized by higher mobility and improved ability to fill the gaps and porosities of the perfect and stable surface of the treated adherends, establishing new contact points and enhancing the interfacial strength due to the mechanical anchoring mechanisms.

#### 3.4.3. Fractographic Analysis of Various Single Lap Joints

**Figure 10:**
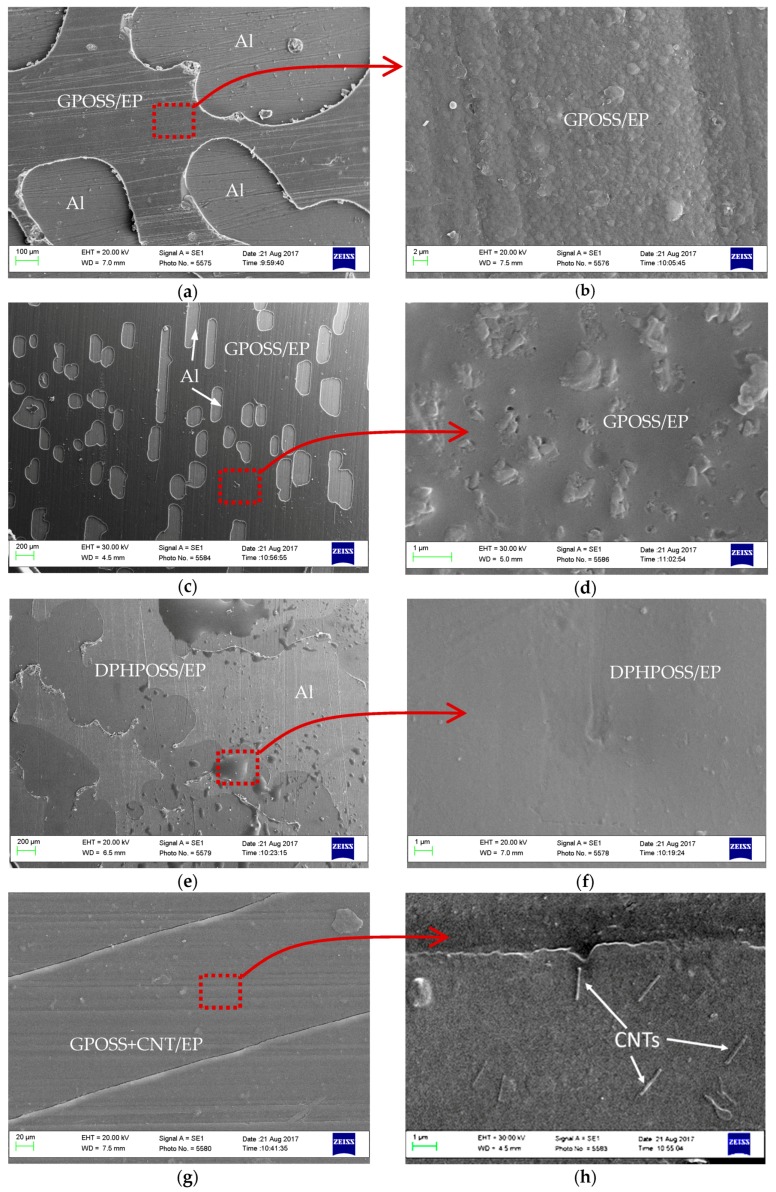
Fractographic analysis of fracture surfaces in the single lap joints (SLJs) with various epoxy nanocomposites: (**a**) Treated Al with 5 wt % GPOSS; (**b**) Treated Al + 5 wt % GPOSS; (**c**) Untreated Al + 5 wt % GPOSS; (**d**) Untreated Al + 5 wt % GPOSS; (**e**) Treated Al—5 wt % DPHPOSS; (**f**) Treated Al—5 wt % DPHPOSS; (**g**) Treated Al—5 wt % GPOSS + 0.5 wt % CNT; (**h**) Treated Al- 5 wt % GPOSS + 0.5 wt % CNT.

SEM analyses were carried out on fracture surfaces of the various lap joints. Micrographs Figure 10a–h show samples of the fracture surface topography of untreated aluminium and CAA-treated aluminium adherends from SLJ made with various epoxy nanocomposites. In Figure 10a,b the fracture surface of treated aluminium adherend of SLJ bonded with 5 wt % GPOSS/EP is shown; while in Figure 10c,d, fracture surface of untreated aluminium adherend of SLJ bonded with 5 wt % GPOSS/EP is shown. Compared to the untreated surface, the CAA-treated surface is much more pitted. In Figure 10e,f fracture surface of treated aluminium adherend of SLJ bonded with 5 wt % DPHPOSS/EP is shown. In Figure 10g,h fracture surface of treated aluminium adherend of SLJ bonded with 5%, GPOSS + 0.5% CNT/EP, which was failed cohesively, is shown. These images confirm the good dispersion of CNTs, achieved by mixing the CNTs in the epoxy resin for a long time, due to the decrease in viscosity caused by the GPOSS incorporation; hence, the agglomerated structure has been broken down and uniformly dispersed.

It is worth noting that the surface of the adherend in Figure 10g is uniformly covered by an adhesive layer. The visual observation highlights that both sides of the adherents are covered by adhesive. Furthermore, the inclusion of MWCNTs into the epoxy adhesive containing GPOSS improves mechanical strength of the adhesive layer; it effectively transfers the external load to the adhesive layer containing nanotubes and the failure occurs cohesively in the domains containing nanotubes, which behave as the strongest part of the composite adhesive. In the same zones, few CNTs are even pulled out of the resin. In fact, they are partly inside the resin (not visible) and partly are out of resin (the visible part) (see MWCNTs marked with arrows). In this regard, it is worth noting that no etching procedure was performed on the fracture surface before the morphological investigation. It is well known in the literature that the morphological investigation, by means of SEM investigations of nanofillers inside polymeric matrix, requires pre-treatment of sample surfaces with strong etching procedure. The simple fact of observing some nanotubes so clearly (see Figure 10h) evidences that they have endured a load which has caused their detachment and pulled-out from the matrix. This is clear evidence that the failure of joint occurs in a cohesive mode. A further support for this hypothesis is provided by the difference in the geometrical parameters observed for MWCNTs alone and MWCNTs pulled-out from the resin in Figure 10h.

As shown in Figure 1 of the Section 2.1. “Materials for adhesive formulations”, the diameter of MWCNTs ranges from 10 nm to 30 nm. In Figure 10h, the diameter of MWCNTs which are partly inside the resin and partly pulled-out from the resin ranges between 120 nm and 160 nm (considering the visible part). The higher values observed for the diameter of MWCNTs in Figure 10h, with respect to the values observed in Figure 1, are a clear indication that the part of carbon nanotube which is out is coated with a layer of resin with an estimated thickness of 40–70 nm. This last observation highlights a very effective micro bridging function of CNTs across the fracture surface which can explain, in a very convincing way, the reason for the relevant enhancement in LSS of the GP-CNT/EP specimen in comparison with GP/EP specimen (see Figure 9).

## 4. Conclusions

In this study, we investigated the effect of carbon nanotubes and two differently functionalized POSS compounds, Glycidyl Oligomeric Silsesquioxanes (GPOSS) and DodecaPhenyl Oligomeric Silsesquioxanes (DPHPOSS), on the adhesion properties of an epoxy resin based on the TGMA precursor, which is particularly suitable for aeronautical applications. The effects of the different chemical functionalities of POSS, as well as the synergetic effect of combination of the CNT and POSS combination on adhesion strength, were evaluated by viscosity measurement, tensile tests, DMA analysis, and morphological investigation. Bonded single-lap joints were made using both untreated and Chromic Acid Anodisation (CAA)-treated aluminium alloy T2024-T3 adherends. Single lap joint shear tests were conducted to measure the lap shear strength of the adhesive bond.

Evaluation of the viscosity at temperatures close to the curing temperature highlighted the beneficial effect that the GPOSS contributed to the formation of the less rigid cross-linked phase, which led to a lower viscous formulation counterbalancing the effect of CNTs in increasing the viscosity. On the contrary, the addition of the non-reactive and insoluble DPHPOSS to the epoxy formulation containing MWCNT caused a slight increment in the viscosity, similar to the case where inert solid micro/nano-fillers were added to polymer matrices, indicating lack of even weak interactions between DPHPOSS and the epoxy matrix and MWCNT.

Tensile tests of the nanocomposite resins showed that the incorporation of insoluble DPHPOSS in the neat epoxy system and in the formulation containing MWCNT did not cause any appreciable differences in the values of Young’s modulus and the tensile strength. On the contrary, the introduction of GPOSS increased all the mechanical characteristics of resin and this effect was ascribed to the formation of a less rigid continuous phase, which caused an improvement of the properties at break of the brittle epoxy matrix.

The dynamic mechanical analysis performed on cured samples confirmed this hypothesis. In fact, the results for GP-CNT/EP samples which contain both GPOSS and MWCNT showed the presence of an additional tan δ peak at a lower temperature, indicating the formation of a higher mobility phase. This additional tan δ peak was, instead, not observable in the DMA test performed on sample DPHP-CNT/EP containing both DPHPOSS and MWCNT.

Single lap joint shear strength tests on specimens made with untreated adherends demonstrated that DPHPOSS caused an improvement in the adhesion properties on the substrate of the adherends. This was attributed to a combined effect of the formation of the electron acceptor-free metallic ions Mg^2+^ on the surface of the adherends being able to interact with the more electronegative oxygen at the bridge between two Si in the POSS cage with the phenyl groups of the DPHPOSS. These interactions caused an improvement in the wettability and in the adhesion between the DPHPOSS-containing epoxy adhesive and the aluminium alloy.

Single lap joint shear strength tests also performed on joints made with CAA-treated adherends provided results with a completely different trend. The best results were obtained for the adhesive sample GP-CNT/EP, while no improvement, compared to the neat epoxy system, was observed for samples containing DPHPOSS. The Alodine chromic acid anodization of the adherends caused a substantial improvement of the adhesion properties of the GP-CNT-modified epoxy, whose adhesive composition included both GPOSS and MWCNTs. This important improvement was attributed to the combination of different phenomena: (i) the development of a stable surface of the adherends with a very homogeneous porosity leading to a strong Al/modified epoxy interphase; (ii) the improved dispersion of MWCNTs in the matrix due to the decrease in viscosity caused by the GPOSS incorporation; (iii) the formation of a phase with higher mobility governed by hydrogen bonds; and, most of all, (iv) a very effective micro bridging function of CNTs across the fracture surface. This last peculiar behavior of MWCNTs in the adhesive formulation provides a very convincing explanation of the relevant enhancement in Lap Shear Strength detected for the GP-CNT/EP specimen in comparison with the GP/EP specimen.

For the CAA-treated aluminium substrate joined with the epoxy DPHPOSS adhesive, instead, the beneficial effect due to the formation of a stable oxide layer was nullified by the complete removal of the magnesium ions from the surface of the adherends.

## Figures and Tables

**Table 1:** Polyhedral Oligomeric Silsesquioxane (POSS) compounds used in this study.

Acronym	Configuration	Characteristics
Glycidyl Oligomeric Silsesquioxanes (GPOSS)	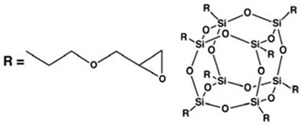	(1) Appearance: viscous liquid
		(2) Color: colorless to slightly yellow
		(3) Molecular/chemical formula: (C_6_H_11_O_2_)*_n_*(SiO_1.5_)*_n_* *n* = 8, 10, 12
		(4) Molecular weight: 1337.88 FW
DodecaPhenyl Oligomeric Silsesquioxanes (DPHPOSS)	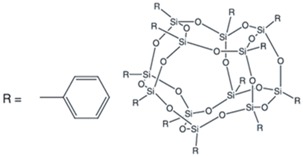	(1) Appearance: powder
		(2) Color: white
		(3) Molecular/chemical formula: C_72_H_60_O_18_Si_12_
		(4) Molecular weight: 1550.26 FW

**Table 2:** Adhesive compound.

Adhesive Composition	Epoxy Blend wt %	MWCNT wt %	GPOSS wt %	DPHPOSS wt %
Epoxy	100	-	-	-
CNT/EP	99.5	0.5	-	-
GP/EP	95	-	5	-
GP-CNT/EP	94.5	0.5	5	-
DPHP/EP	95	-	-	5
DPHP-CNT/EP	94.5	0.5	-	5

**Table 3:** Summary of the prepared single lap joint samples.

Sample Label	Adherend	Adhesive Composition	Adhesive Thickness [mm]
Al-Epoxy	Aluminium	Epoxy	0.16 ± 0.02
Tr-Al-Epoxy	Treated aluminium	Epoxy	0.18 ± 0.03
Al-GP	Aluminium	GP/EP	0.20 ± 0.01
Tr-Al-GP	Treated aluminium	GP/EP	0.21 ± 0.01
Al-DPHP	Aluminium	DPHP/EP	0.19 ± 0.01
Tr-Al-DPHP	Treated aluminium	DPHP/EP	0.20 ± 0.01
Al-GP-CNT	Aluminium	GP-CNT/EP	0.23 ± 0.05
Tr-Al-GP-CNT	Treated aluminium	GP-CNT/EP	0.22 ± 0.02
Al-DPHP-CNT	Aluminium	DPHP-CNT/EP	0.25 ± 0.05
Tr-Al-DPHP-CNT	Treated aluminium	DPHP-CNT/EP	0.25 ± 0.03

**Table 4:** Measured viscosity η * [Pa s] at different temperatures for pristine epoxy resin and filled formulations.

Temperature (°C)	Epoxy η (Pa·s)	CNT/EP η (Pa·s)	GP-CNT/EP η (Pa·s)	DPH-CNT/EP η (Pa·s)
80	0.40	7.00	3.11	7.5
90	0.11	5.00	1.34	5.6
100	0.10	3.50	0.65	4.9
110	0.07	2.10	0.35	5.0 *
120	0.05	3.05 *	0.22	6.9 *

* start curing.
